# Impending Danger: Evolution of Hyperacute T Waves to Cardiac Arrest

**DOI:** 10.7759/cureus.96989

**Published:** 2025-11-16

**Authors:** Christopher Poyorena, Christopher Allen, Lauren B Querin

**Affiliations:** 1 Emergency Medicine, University of Washington Harborview Medical Center, Seattle, USA; 2 Emergency Medicine, Stanford University, Palo Alto, USA; 3 Department of Emergency Medicine, Mayo Clinic Arizona, Phoenix, USA

**Keywords:** anabolic androgenic steroid, anginal chest pain, hyperacute t waves, st-elevation myocardial infarction (stemi), stemi equivalent

## Abstract

Acute coronary syndrome (ACS) is a leading cause of cardiac arrest, and although historically seen in an older patient population, recent decades have shown an increase in prevalence among younger individuals. Emergency reperfusion or percutaneous coronary intervention (PCI) has been the standard of care for patients with electrocardiograms (ECGs) that meet the criteria for ST-elevation myocardial infarction (STEMI). Several ECG STEMI equivalents have also been introduced and implemented in emergency cardiac care, including the presence of hyperacute T waves. This case describes a 45-year-old previously healthy male with a history of anabolic steroid and pre-workout supplementation use who presented with chest pain. Initial ECG showed hyperacute T waves, and the patient’s clinical status rapidly deteriorated, leading to ventricular fibrillation and cardiac arrest. Post-return of spontaneous circulation, the patient’s ECG met clear STEMI criteria. Emergency physicians must be familiar with standard STEMI criteria, as well as STEMI equivalents, in order to appropriately involve interventional cardiology consultants early for consideration of coronary reperfusion. Emergency physicians should also maintain a higher clinical suspicion for ACS in individuals with anabolic steroid or unregulated pre-workout supplements.

## Introduction

Acute coronary syndrome (ACS) is a leading cause of both in- and out-of-hospital cardiac arrest [1,2]. ACS-related cardiac arrest is most commonly associated with ST-elevation myocardial infarction (STEMI) [3,4]. Historically, percutaneous coronary intervention (PCI) capable centers have leaned heavily on the presence of STEMI on the electrocardiogram (ECG) for activation criteria. This practice has come under question in recent decades after studies have shown up to 40% of complete occlusive acute myocardial infarctions (AMI) were missed when using the presence of STEMI on ECG alone as the main decision point for PCI, leading to detrimental delays in reperfusion therapy [5]. The American College of Cardiology (ACC) 2022 expert consensus review recognized that, in addition to STEMI, multiple “STEMI equivalents” suggest either AMI or imminent AMI and warrant an emergent evaluation for PCI. These ECG patterns include posterior STEMI, presence of Sgarbossa criteria or Smith-modified Sgarbossa criteria in the setting of a ventricular-paced rhythm or left bundle branch block, de Winter sign, and hyperacute T waves [6].

Although historically ACS has been seen predominantly in the older adult population (age >65 years), there has been a significant trend over the recent decades of ACS in younger patients, attributed in large part to modifiable risk factors [7]. We report a case of a young patient with a unique cardiac risk factor presenting for chest pain and was found to have hyperacute T waves on initial ECG, followed by rapid decompensation to cardiac arrest. 

## Case presentation

A previously healthy 45-year-old male presented to the emergency department (ED) with intermittent, substernal chest pain radiating down his left arm over the prior two days. His symptoms first began a few hours after using a new pre-workout supplement and weightlifting at the gym. They persisted for a couple of hours and self-resolved. His pain returned the day of presentation under the same conditions, although more persistent, which prompted his evaluation. Associated symptoms included shortness of breath, nausea, and emesis. He denied pleuritic pain, extremity edema, hemoptysis, syncope, fever, cough, or trauma. On further history, he reported prior use of anabolic steroids, with the last being six weeks prior to presentation. It was unclear what the contents of the pre-workout supplement were, but he denied any tobacco or illicit drug use. Notably, he had a family history of heart disease, with his father having undergone coronary bypass at age 47.

On initial evaluation, the patient was slightly hypertensive with a blood pressure of 161/107 mmHg and with otherwise normal vital signs. Physical examination revealed a diaphoretic male in moderate distress, but alert and interactive. He had non-labored breathing and a normal cardiopulmonary exam. He had no focal neurologic deficits and no peripheral edema. Distal extremity pulses were palpable and equal bilaterally.

**Figure 1:**
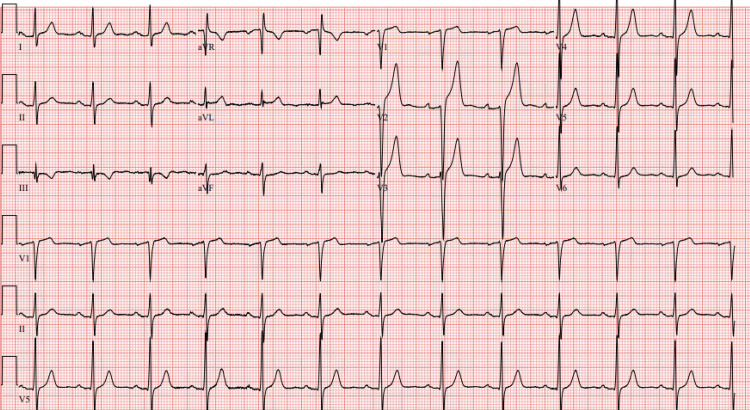
Initial emergency department (ED) electrocardiogram (ECG) demonstrates hyperacute T waves in leads V2-4 with ST elevation in leads V2-V3 without reciprocal depression.

Initial work-up was notable for a mild polycythemia with a hemoglobin of 19.0 g/dL (ref. range: 13.5-17.5 g/dL) and a high-sensitivity Troponin T (5th generation) of 97 ng/L (ref. range: <=15 mg/L). The initial ECG is depicted in Figure 1 and shows ST elevations in V2-V3 with tall, broad-based, asymmetric T waves in V2-V4 with no reciprocal ST depressions.

**Figure 2:**
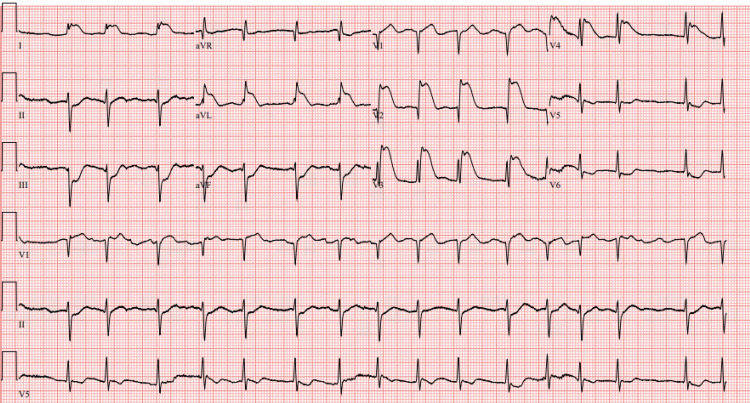
Post-arrest electrocardiogram (ECG) demonstrates marked concave ST elevations in the anterolateral leads with reciprocal ST depressions in the inferior leads, meeting ST-elevation myocardial infarction (STEMI) criteria.

Unfortunately, just 15 minutes after the initial ECG was obtained, the patient went into cardiac arrest with a ventricular rhythm. He underwent immediate advanced cardiovascular life support (ACLS), including defibrillation. During the code, he was intubated and sedated. After the second defibrillation, return of spontaneous circulation (ROSC) was obtained. Post-arrest ECG, obtained 45 minutes after the initial (Figure 1), showed marked ST elevations in the anterior leads and depressions in inferior leads consistent with anterior STEMI (Figure 2).

**Figure 3:**
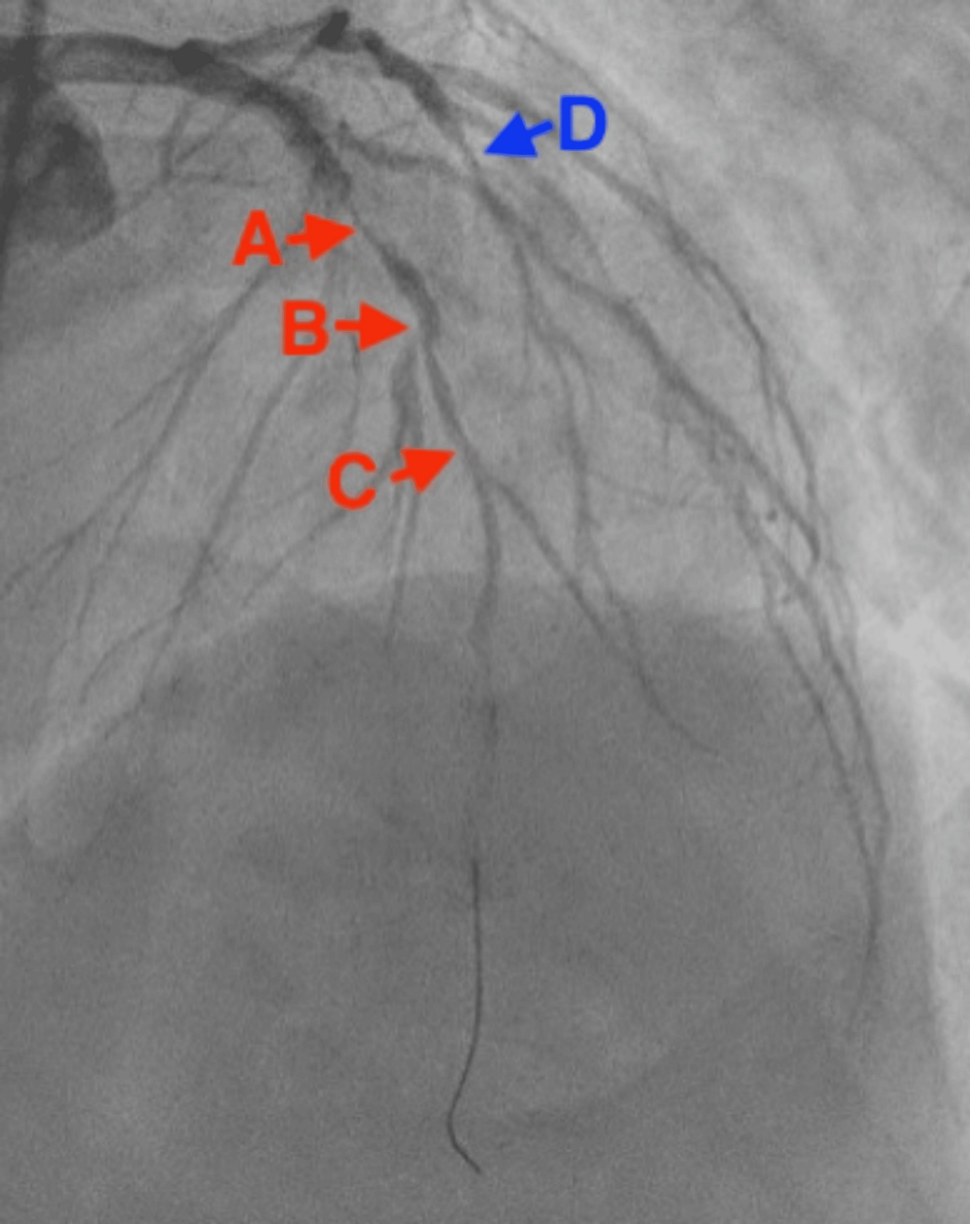
Cardiac catheterization report demonstrating a 100% obstruction (A), a 30% obstruction (B), and a 60% obstruction (C) of the left anterior descending (LAD) artery, as well as a 30% obstruction of the circumflex artery.

The patient was taken emergently to PCI and was found to have a complete occlusion of the left anterior descending artery (LAD) as well as partial obstruction(<50%) of the circumflex artery (Figure 3). Four drug-eluting stents (DES) were placed.

Cardiac risk laboratory evaluation also showed elevated serum triglycerides of 213 mg/dL (normal reference: <150 mg/dL) and low serum HDL of 25 mg/dL (normal reference >= 40 mg/dL). Hemoglobin A1c was normal. Post-procedural care was complicated by initial hypotension, transiently treated with dopamine and norepinephrine drips, and an echocardiogram showing a reduced left ventricular ejection fraction (LVEF) of 28% with anterior wall motion abnormalities. The patient was discharged neurovascularly intact on hospital day 4 with a repeat echocardiogram showing a persistent anterior wall motion abnormality but improved LVEF of 43%.

## Discussion

This patient’s case demonstrates how hyperacute T waves may be initially the only ECG finding in a patient with AMI and how clinical status can rapidly progress to cardiac arrest, even without ECG progression to a classic STEMI pattern. Hyperacute T waves on ECG are defined as broad, asymmetrically peaked T waves in at least two contiguous leads [6]. The physiologic mechanism causing the hyperacute morphology on ECG is related to ischemia-induced changes in potassium currents, resulting in accelerated repolarization of the epicardium compared to the endocardium, reflected as increased T wave amplitude [8,9]. Hyperacute T waves can represent early cardiac ischemia and may rapidly evolve in the span of minutes to classic STEMI findings on ECG, underscoring the importance of obtaining serial ECGs.

Hyperacute T waves remain a diagnostic challenge as they have not been clearly defined in the literature and are often difficult to differentiate from normal variants, hypertrophy, or hyperkalemia [10-12]. A recent study attempted to quantify myocardial ischemia-induced hyperacute T-waves as a T-wave amplitude that exceeds the 95th percentile of normal; however, results showed this was unreliable in predicting ischemia in leads other than III, aVR, and V1 [13]. Due to this uncertainty, the ACC recommends obtaining serial ECGs and involving interventional cardiology early for consideration of PCI [6].

From the early 2000s forward, the number of younger patients, aged 35-54 years, diagnosed with ACS has increased substantially [14]. This trend is thought to be related to the use of illicit drugs, e-cigarette use, increased sedentary lifestyles, and the rise of stimulant exercise enhancement substances and anabolic steroid use [14]. Both androgenic-anabolic steroids (AAS) and pre-workout drinks have been known to increase the risk of CAD by increasing lipoprotein production, causing arterial intimal hyperplasia, increasing clotting factors leading to a procoagulant state, and increasing other cardiovascular risk profiles such as hypertension [15-17]. The consensus on the adverse effects and overall safety of pre-workout supplements remains under debate as they are unregulated by the Food and Drug Administration. Several studies have found preworkout use to be associated with ACS, arrhythmia development, cardiac syncope, stroke, and many other vascular emergencies [16-17]. 

This case highlights the importance of emergency physicians to inquire about and maintain a higher level of suspicion for ACS in young patients with the use of androgenic-anabolic steroids or stimulant exercise enhancement agents. It also emphasizes the need for rapid interval serial ECGs and involvement of interventional cardiology early in cases of chest pain with hyperacute T waves on ECG. Although current literature supports the interpretation of hyperacute T waves as a STEMI-equivalent or at minimum raising concern for AMI, it is important to note that there remains no formal consensus that defines the ECG criteria for hyperacute T waves [7]. As a result, emergency physicians and cardiology consultants are left with vague and variable interpretations, potentially impeding definitive care for patients. This case calls for further research devoted to determining outcome definitions for hyperacute T waves. 

## Conclusions

Using the presence of STEMI on ECG to activate PCI in ACS is missing a large portion of AMI patients who are at high risk for sudden cardiac death. Emergency physicians must embrace new ECG STEMI equivalents when evaluating chest pain in the ED. Hyperacute T waves are considered a STEMI equivalent and can be a harbinger of a developing AMI. Emergency physicians should scrutinize hyperacute T-waves, especially in the setting of chest pain, while also recognizing that their definition remains elusive at this time. Rapid interval serial ECGs and clinical reassessment are essential in these cases.

Patients are also presenting younger with ACS in large part due to modifiable risk factors. Emergency physicians must have an elevated clinical suspicion for ACS when a patient reports use of anabolic steroids, stimulant energy drinks, and marketed as pre-workout supplements.
